# Outcomes of Surgical Versus Percutaneous Peritoneal Dialysis Catheter Insertion Techniques: A Single-Center Experience

**DOI:** 10.7759/cureus.83113

**Published:** 2025-04-28

**Authors:** Manjuri Sharma, Suresh Singh, Prodip K Doley, Gayatri Pegu, Miranda Pegu

**Affiliations:** 1 Nephrology, Gauhati Medical College, Guwahati, IND

**Keywords:** end-stage renal disease, percutaneous, peritoneal dialysis catheter, peritonitis, surgical minilaparotomy

## Abstract

Background

Continuous ambulatory peritoneal dialysis (CAPD) is a feasible and practical option for renal replacement therapy (RRT) in patients with end-stage renal disease (ESRD). However, the superiority of the surgical method versus the percutaneous method for peritoneal dialysis catheter (PDC) placement is not well established.

Methods

We retrospectively analyzed 91 peritoneal dialysis (PD) catheters inserted using two methods: the minilaparotomy technique performed by a surgeon (Group S, n=57) and the percutaneous technique performed by a nephrologist (Group N, n=34) over a 36-month study period.

Results

The primary PDC nonfunction rate was comparable between the two groups (3.5% vs. 3.3%). Catheter survival at one year (78.9% vs. 80%, p=0.761) and at the end of the study (61.4% vs. 66.6%, p=0.947) was higher in Group N but not statistically significant. The mean duration of catheter survival (in months) was identical in both groups (19.62±10.42 vs. 19.62±10.42), and patient survival at the end of the study was also comparable (78.9% vs. 80%, p=0.852). Peritonitis rates (per patient-year) did not differ significantly between the groups (0.15 vs. 0.10, p=0.693). Mechanical complication rates and refractory peritonitis rates were also comparable between the two groups.

Conclusion

The outcomes of percutaneously placed PDCs performed by a well-trained nephrologist were comparable to those placed by surgeons using the minilaparotomy technique. Training more nephrologists in percutaneous PDC insertion could enhance patient access and convenience in care.

## Introduction

Continuous ambulatory peritoneal dialysis (CAPD) is a feasible and practicable home-based modality of renal replacement therapy (RRT) for end-stage renal disease (ESRD) patients, especially in cases of difficult access to hemodialysis centers [1,2]. Peritoneal dialysis catheter (PDC) insertion success is a critical component of a successful CAPD program. Surgeons commonly use either the open surgical (minilaparotomy) or the laparoscopic technique. However, issues like the requirement of an operation theatre (OT), an anesthetist, clearances for surgery, and scheduling issues may cause delays and prolonged hospital stays [3].

Nephrologists who implant PDC percutaneously report similar outcomes to those who implant them surgically, as shown by Ozener et al. [4] and metanalysis published [5]. We conducted a study comparing the outcomes of recently initiated PDC insertions performed by nephrologists with those performed by surgeons using the minilaparotomy technique at our center. The primary objective was to compare the rate of primary nonfunction of PDCs inserted by surgeons versus those inserted by nephrologists. The secondary objective was to compare catheter survival, patient survival, and complication rates between the two groups.

## Materials and methods

This was a retrospective study conducted in the Department of Nephrology, Gauhati Medical College, Guwahati, Assam. All ESKD patients aged >12 years who were eligible for RRT initiation during the study period from January 2021 to December 2023 were considered. The study was conducted between January 2021 and December 2023, with a recruitment period of two years (January 2021 to December 2022) and a one-year follow-up period. Ethical clearance was obtained from the Institutional Ethics Committee before starting the study.

All ESKD patients visiting the Nephrology outpatient department (OPD) who required RRT initiation during the recruitment period (January 2021 to December 2022) were included. All surgical minilaparotomy CAPD catheter insertions performed by surgeons and all percutaneous CAPD catheter insertions performed by nephrologists were included. Patients were aged >12 years, of either sex, and were expected to remain in the state for at least one year for follow-up. Critically ill patients (those requiring ICU or critical care admission with unstable vitals), those requiring major abdominal surgery (defined as any surgery involving a breach of the peritoneum, excluding laparoscopic procedures like laparoscopic cholecystectomy and laparoscopic appendectomy), patients who underwent concurrent PDC implantation along with abdominal hernia repair or other abdominal surgeries, and those with morbid obesity (body mass index (BMI) >30 kg/m²) were excluded.

A total of 91 patients underwent PDC insertion during the study period in the Department of Nephrology at our institute. Data were collected from patient records and surgical operation notes. Depending on supply availability at our center and patient preference, two types of PDCs were used: Swan-neck PDC with a straight tip, and Tenckhoff straight PDC with either a straight or coiled end. Following PDC insertion, after a break-in period of six days, patients in both study groups received training for CAPD initiation. After CAPD initiation, patients were followed up in the Nephrology OPD at one month for peritoneal equilibration testing (PET), or earlier in case of any PD-related issues. Subsequent follow-up was done every two months, either physically in the Nephrology OPD or via telephone, depending on clinical needs and physician discretion. The definitions used in this study were based on the ISPD peritonitis guideline recommendations (2020 and 2022 updates).

Outcomes

The percentage of PDCs removed due to primary nonfunction in the surgical and percutaneous groups was the primary immediate outcome. Primary nonfunction was defined as a catheter that failed either immediately after insertion or at a later stage, rendering it impossible to perform CAPD exchanges before removal. Long-term outcomes included patient survival, PDC survival, pre-PD peritonitis, catheter infection rate (tunnel infection or exit-site infection), PDC insertion-related peritonitis, overall peritonitis rate, and peri-catheter leak.

Data collection

Data collected included the date of catheter insertion, indication for CAPD, whether insertion was performed by a surgeon or nephrologist, operative records, type of catheter used, immediate postoperative catheter position, and any mechanical or non-mechanical complications post-insertion. The functional status of the PDC during the initial flushing was also recorded. Information regarding the underlying disease and comorbidities was obtained from OPD and inpatient medical records. Details regarding catheter survival, number of peritonitis episodes, outcomes of these episodes, date and cause of catheter removal, reasons for CAPD failure, and patient survival were obtained during OPD follow-ups or via telephonic inquiries. Each catheter insertion was considered a separate event if a patient underwent PDC insertion more than once. All data were recorded in SPSS.

Catheter placement procedure

Surgeons minilaparatomy technique: PDCs were predominantly placed by two surgeons using an open surgical technique. In the main operating room, under local anesthesia and mild sedation, a mini-laparotomy was performed. The procedure involved a vertical paramedian incision (2-3 cm in length, lateral to the midline), followed by dissection of subcutaneous abdominal layers and hemostasis. The anterior rectus sheath was incised, rectus muscle fibers were retracted, and the posterior rectus sheath was opened. A small nick was made in the parietal peritoneum to allow passage of the PDC. After confirming entry into the peritoneal cavity, the PDC was introduced and directed toward the contralateral iliac fossa. The internal cuff was placed in the pre-peritoneal space and sewn to the parietal peritoneum. Both rectus sheaths were then closed, and the catheter was brought out through a subcutaneous tunnel, with the external cuff positioned approximately 2-3 cm from the exit site.

Nephrologist’s percutaneous technique: Nephrologists used a modified Seldinger technique with a catheter insertion kit (Medcom), under local anesthesia. After identifying and avoiding the inferior epigastric artery using color Doppler, a vertical paramedian incision was made 3-4 cm from the midline, just medial to the lateral border of the rectus muscle. Following the skin incision, blunt dissection was performed to reach the anterior rectus sheath. Using the introducer needle from the PDC kit and under ultrasound guidance, the needle was inserted into the peritoneum, aiming toward the opposite iliac fossa. Entry into the peritoneal cavity was confirmed by the characteristic "give-away" sensation and aspiration using a 10 mL syringe. Then, 500 mL of dialysate fluid was instilled to prime the cavity and prevent bowel injury.

A peel-away sheath with an introducer was advanced over a guidewire, which had been inserted through the needle. After guidewire removal, a straight, double-cuffed, coiled-end Tenckhoff catheter was inserted through the sheath using a stylet aimed at the opposite iliac fossa. The internal cuff was positioned between muscle layers and secured at the anterior rectus sheath. Intraoperatively, 500 mL of 2.5% PD fluid was instilled through the catheter to confirm proper inflow and outflow. A subcutaneous tunnel was created caudolaterally, maintaining a 2 cm distance from the exit site to the external cuff. The main procedural differences between the surgical and nephrologist techniques are summarized in Table 1.

**Table 1:** Comparison of PDC insertion procedures between surgical minilaparotomy techniques and nephrologist-performed percutaneous methods PDC, peritoneal dialysis catheter; OT, operation theatre

Variables	Group S	Group N
Operators	surgeon‑1 + 1 assistant	Nephrologist‑1+ 1 assistant
Place of insertion	Minor OT	Bedside/minor OT
Anesthesia	Sedation + local	Sedation + local
Skin incision	4‑5 cm, paramedian	2‑3 cm, paramedian
Inner cuff location	Pre-peritoneal space and sutured with posterior rectus sheath	In between the rectus muscle, sutured to anterior rectus sheath
USG guidance for introducer needle into cavity	No, as bowels can be directly visualized	Yes
Instillation of cavities before catheter insertion	No, as bowels can be directly visualized	Yes
Intra‑operative testing of catheter function	No	Yes
Percutaneous tunnel	10‑12 cm, caudolateral	10‑12 cm, caudolateral
Time period of post-insertion ambulation	48 h	12 h

Statistical analysis

Data were coded and recorded in SPSS version 23 (IBM Corp., Armonk, NY) and used for analysis. Descriptive statistics were presented as means with standard deviations or medians with interquartile ranges (IQRs) for continuous variables and as frequencies and percentages for categorical variables. A two-tailed p-value of <0.05, determined using the chi-square test, was considered statistically significant. Kaplan-Meier curves were generated for survival analysis.

## Results

**Figure 1:**
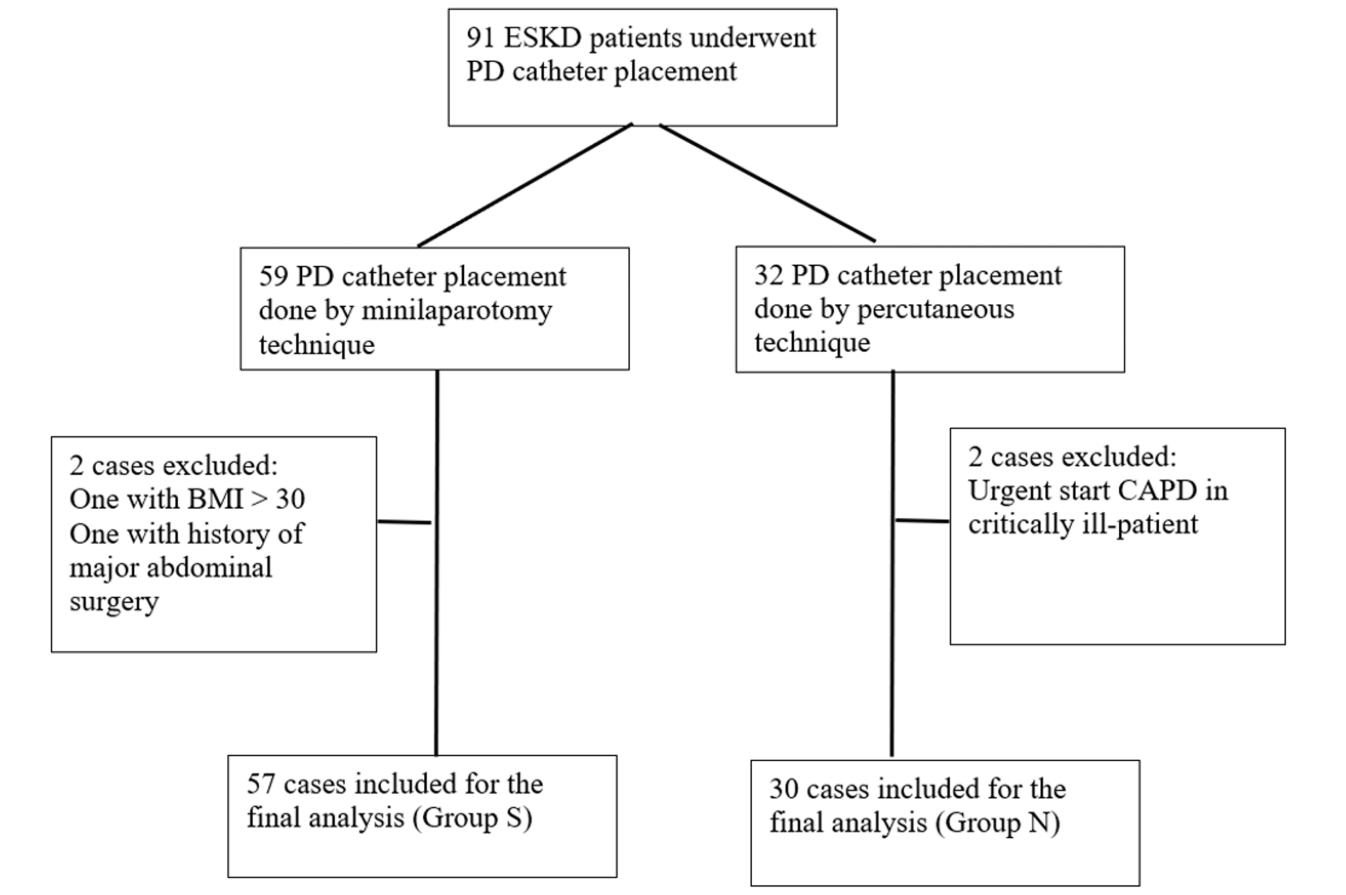
Total PDC performed and final number included for analysis PDC, peritoneal dialysis catheter; PD, peritoneal dialysis; CAPD, continuous ambulatory peritoneal dialysis

A total of 91 PDC insertions were performed; four were excluded, and the remaining 87 insertions, performed in 85 patients, were analyzed. Two patients in Group S underwent immediate re-insertion of the CAPD catheter due to malposition of the PDC tip. Of the 87 analyzed PDC insertions, 57 were performed by surgeons (Group S) and 30 by nephrologists (Group N) (Figure 1). Both the groups were comparable regarding the baseline characteristics (Table 2).

**Table 2:** Baseline characteristics of the study population Data represented as n (%) and mean±SD; p<0.05 is considered significant. ^#^Independent sample t-test (t-value) ^$^Chi-square test (chi-square value) PDC, peritoneal dialysis catheter

Parameters	Group S (n=57)	Group N (n=30)	Test value	P-value
Patients included in the final analysis (n)	55	30		
Number of PDCs (n)	57	30		
Mean age (years)	55.7±14.2	52.2±12.2	0.454^#^	0.234
Male sex	32 (56.1%)	18 (60.0%)	0.647^$^	0.301
Female sex	25 (43.8%)	12 (40.0%)		
Co-morbidities				
Diabetes mellitus	22 (38.5%)	13 (43.3%)	0.873^$^	0.178
CAD	7 (12.2%)	5 (16.6%)		
Liver disease	1 (1.75%)	3 (10.0%)		
Prior minor abdominal surgery	4 (7.0%)	2 (6.6%)		
Indications for CAPD				
Patient preference	41 (71.9%)	19 (63.3%)	0.565^$^	0.254
Vascular access failure	10 (17.5%)	6 (20.0%)		
Previous PDC failure	2 (3.5%)	0 (0.0%)		
Refractory heart failure	4 (7.0%)	3 (10.0%)		
Intradialytic hypotension	0 (0.0%)	2 (6.6%)		
Catheter type				
Swan neck	31 (54.3%)	10 (33.3%)	1.128^$^	0.062
Tenckhoff straight	26 (45.6%)	20 (66.6%)		

All PDCs inserted by surgeons using the minilaparotomy technique were double-cuffed, Swan-neck, straight-tip catheters (not provided with a sheath/dilator), whereas all PDCs inserted by nephrologists using the percutaneous technique were double-cuffed, coiled-tip catheters (provided with a sheath/dilator), based on the catheter availability at our center.

The primary non-function rate of PDCs in Group S (3.5%) was numerically slightly higher than in Group N (3.3%), but the difference was not statistically significant (P=0.966) (Table 3).

**Table 3:** Comparison of immediate outcome and survival between Group S and Group N Data represented as n (%) and mean±SD; p<0.05 is considered significant. ^#^independent sample t-test (t-value) ^$^Chi-square test (chi-square value) PDC, peritoneal dialysis catheter

Outcome measures	Group S (n=57)	Group N (n=30)	Test value	P-value
Primary PDC non-function rate	2 (3.5%)	1 (3.3%)	0.006^$^	0.966
Catheter survival				
At one year	45 (78.9%)	24 (80%)	0.243^$^	0.761
At the end of the study	35 (61.4%)	20 (66.6%)	0.014^$^	0.947
Duration of catheter survival (months)	19.62±10.42	18.88±10.82	0.237^#^	0.568
Patient survival at the end of the study	45 (78.9%)	24 (80.0%)	0.178^$^	0.852
Death with functional catheter	9 (15.7%)	4 (13.3%)	0.188^$^	0.839

A total of three cases of primary catheter non-function occurred, two in Group S and one in Group N. All three were due to the malposition of the catheter tip, resulting in outflow failure. There was no crossover of patients between the two groups, and the same technique was used for subsequent PDC placement following primary failure in each group. Table 4 presents the long-term outcome measures in both groups.

**Table 4:** Comparison of long-term outcomes between Group S and Group N Data represented as n (%); p<0.05 is considered significant. ^$^Chi-square test (chi-square value) PDC, peritoneal dialysis catheter

Outcome measures	Group S (n=57)	Group N (n=30)	Test value	P-value
Catheter migration	3 (5.2%)	1 (3.3%)	0.173^$^	0.857
Pericatheter leak	1 (1.75%)	1 (3.3%)	0.005^$^	1.000
Hematoma	1 (1.75%)	0 (0.0%)	0.307^$^	0.675
Hemorrhagic outflow	2 (3.5%)	1 (3.3%)	0.025^$^	0.904
Peritonitis rate (per patient-year)	0.15	0.1	0.311^$^	0.693
Exit site infection	2 (3.5%)	0 (0.0%)	0.251^$^	0.732
Causes of PDC removal				
Catheter malfunction	6 (10.5%)	1 (3.3%)	0.534^$^	0.236
Ultrafiltration failure	3 (5.2%)	2 (6.6%)	0.274^$^	0.752
Death	7 (12.2%)	4 (13.3%)	0.164^$^	0.861
Refractory peritonitis	5 (8.7%)	1 (3.3%)	0.335^$^	0.546
Kidney transplantation	1 (1.75%)	2 (6.6%)	0.479^$^	0.455

**Figure 2:**
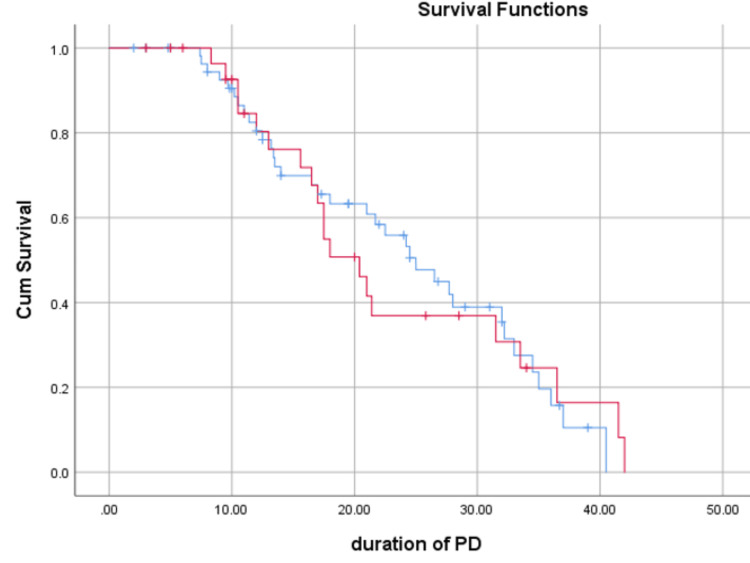
Kaplan-Meier curves for PDC survival (in months) between two groups PDC, peritoneal dialysis catheter; PD, peritoneal dialysis

**Figure 3:**
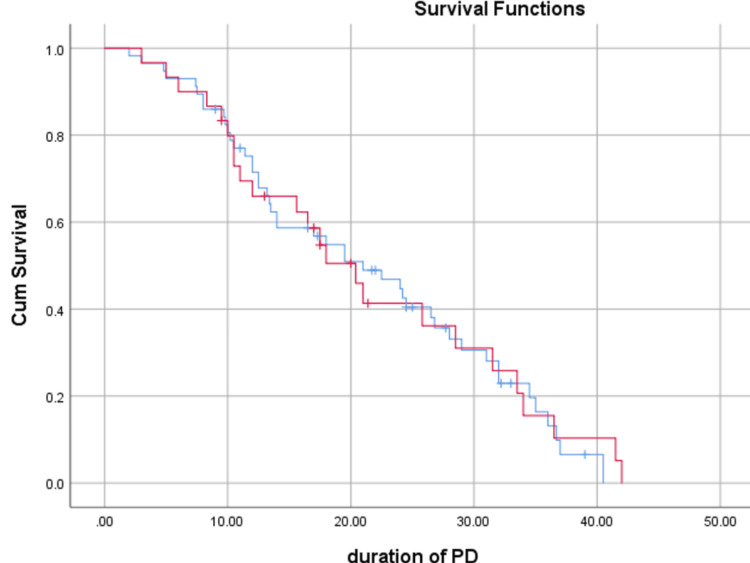
Kaplan-Meier curves for patient survival between the two groups PD, peritoneal dialysis

Figure 2 and Figure 3 of exit site bleeding or peri-catheter leak, exit site infection rate, and peritonitis rate. Following catheter insertion, no patients in either group developed pre-PD peritonitis, bowel injury, or tunnel infection.

## Discussion

The long-term survival of PDCs primarily depends on the expertise of the operator, the insertion technique used, the type of catheter inserted, and the occurrence of mechanical or infectious complications. At our center, all PDC insertions were initially performed using the surgical minilaparotomy technique. However, with the development of in-house expertise, percutaneous PDC insertion was recently introduced. The nephrologists adopted the Seldinger technique, which eliminated the need for an operating theatre, an anesthetist, and related scheduling issues. Since its introduction, the annual number of percutaneous PDC insertions at our center has steadily increased.

Percutaneous PDC insertion is essentially a "blind" technique, lacking direct visualization of the peritoneum. Although generally considered safe, some reports have suggested a higher incidence of leaks and a potential risk of bowel perforation [6,7]. Due to the unavailability of fluoroscopy in our department, we used bedside ultrasound guidance to identify the peritoneal cavity and minimize the risk of bowel injury during catheter placement.

The primary PDC non-function rate was slightly higher in Group S compared to Group N (2 cases, 3.5% vs. 1 case, 3.3%), although this difference was not statistically significant (P=0.966). Similar findings were reported by Sivaramakrishnan et al. [3], where the primary non-function rate was 18.2% (16/88) in the surgical group versus 7.3% (4/55) in the percutaneous group (P=0.08). In contrast, Medani et al. observed a lower primary PDC failure rate in the surgical group compared to the percutaneous group (0.0% vs. 2.6%, P=0.05) [8], which may be attributed to differences in catheter types used, coiled-tip catheters in the surgical group versus straight-tip in the percutaneous group.

Although catheter survival was better in Group N compared to Group S (66.6% vs. 61.4%), the difference did not reach statistical significance (P=0.947). Similar results were reported by Dogra et al., with better PDC survival in the percutaneous group (53.1%) compared to the surgical group (50.5%; P = 0.2) [9].

Our study showed better patient survival and fewer deaths with functional catheters in the percutaneous group, although these differences were not statistically significant. Patient survival was 78.9% in Group S and 80% in Group N (P=0.852), while deaths with functional catheters occurred in 15.7% of patients in Group S versus 13.3% in Group N (P=0.839). Similar findings were reported by Dogra et al. (Group S: 77.4%, Group P: 82.9%; P=0.07) [9] and Ozener et al. (Group S: 32%, Group P: 67%; P=0.001) [4].

Post-insertion catheter migration was comparable between the groups (Group S: 3 cases, 5.2% vs. Group N: 1 case, 3.3%; P=0.693). Shah et al. also reported a higher, though statistically non-significant, rate of migration in the percutaneous group (surgeon: 2.5% vs. nephrologist: 4.2%; P=0.53) [10]. Dogra et al. found similar results (Group S: 2.2% vs. Group P: 8.5%; P=0.049) [9]. Although the differences were not statistically significant, the variation may be attributed to differences in catheter type (straight vs. coiled) and operator experience.

Pericatheter leaks were slightly more frequent in the percutaneous group, though not statistically significant (Group S: 1.75% vs. Group N: 3.3%; P=0.693). These findings align with those reported by Dogra et al. (Group S: 0.0% vs. Group P: 4.2%; P=0.049) [9].

No cases of bowel injury were reported in either group in our study. This is consistent with findings by Dogra et al. [9], while Sampathkumar et al. reported one case of bowel injury (not perforation), which resolved without surgical intervention [11].

The peritonitis rate was lower in the percutaneous group, though the difference was not statistically significant (Group S: 0.15 per patient-year vs. Group N: 0.1 per patient-year; P=0.693). These results are consistent with those of Sivaramakrishnan et al., who reported median peritonitis rates (per 1,000 catheter days) of 1.7 in the surgical group and 1.2 in the percutaneous group (P=0.47) [3]. Meta-analyses by Agarwal et al. [5] and Esagian et al. [12] suggest that early infectious complications are fewer in percutaneous insertions, with no significant differences in mechanical complications compared to surgical insertions.

In our study, surgeons most commonly used double-cuffed Swan-neck PDCs (31 cases, 54.3%), whereas nephrologists predominantly used Tenckhoff straight PDCs (20 cases, 66.6%), although this difference was not statistically significant. Both straight and coiled-tip PDCs were used. Currently, no consensus guidelines exist on choosing between straight and coiled-tip catheters. Meta-analyses have shown mixed results, with some favoring coiled-tip and others favoring straight-tip catheters [13,14]. In a retrospective study by Singh et al., 50 percutaneously placed PDCs (28 coiled-tip, 22 straight-tip) were analyzed. The coiled-tip catheters were associated with fewer early migrations (3.6% vs. 31.8%; OR: 12.6; CI: 1.41-112.39; P=0.02), better one-year technique survival (P=0.07), and comparable peritonitis rates (0.14 vs. 0.11 events per patient-year, respectively) [15].

Overall, both groups in our study showed comparable catheter survival, patient survival, and peritonitis rates. Limitations of our study include its retrospective design, a limited follow-up period of one year, and the exclusion of patients with surgically complex abdomens. A prospective study with a larger sample size and longer follow-up (2-3 years) is needed to more accurately assess long-term catheter survival and complication rates.

## Conclusions

The outcomes of percutaneously placed PDCs by well-trained nephrologists were comparable to those achieved by surgeons using the minilaparotomy technique. Both types of PDCs, straight-tip and coiled-tip, demonstrated similar results. Percutaneous placement enables early patient ambulation and, due to its ease of execution compared to surgical minilaparotomy, is a viable option for bedside insertion and urgent-start PD in selected patients. More nephrology departments should consider adopting the percutaneous PDC insertion technique for the benefit of patients with ESKD.

## References

[1] Agarwal SK, Srivastava RK (2009). Chronic kidney disease in India: challenges and solutions. Nephron Clin Pract.

[2] Rajapurkar MM, John GT, Kirpalani AL (2012). What do we know about chronic kidney disease in India: first report of the Indian CKD registry. BMC Nephrol.

[3] Sivaramakrishnan R, Gupta S, Agarwal SK, Bhowmik D, Mahajan S (2016). Comparison of outcomes between surgically placed and percutaneously placed peritoneal dialysis catheters: a retrospective study. Indian J Nephrol.

[4] Ozener C, Bihorac A, Akoglu E (2001). Technical survival of CAPD catheters: comparison between percutaneous and conventional surgical placement techniques. Nephrol Dial Transplant.

[5] Agarwal A, Whitlock RH, Bamforth RJ (2021). Percutaneous versus surgical insertion of peritoneal dialysis catheters: a systematic review and meta-analysis. Can J Kidney Health Dis.

[6] Nicholson ML, Donnelly PK, Burton PR, Veitch PS, Walls J (1990). Factors influencing peritoneal catheter survival in continuous ambulatory peritoneal dialysis. Ann R Coll Surg Engl.

[7] Apostolidis NS, Panoussopoulos DG, Manouras AJ, Pararas BN, Voudiklari SG, Zirogiannis PN (1998). The use of TWH catheters in CAPD patients: fourteen-year experience in technique, survival, and complication rates. Perit Dial Int.

[8] Medani S, Shantier M, Hussein W, Wall C, Mellotte G (2012). A comparative analysis of percutaneous and open surgical techniques for peritoneal catheter placement. Perit Dial Int.

[9] Dogra PM, Hooda AK, Shanmugraj G, Pramanik SK (2018). Continuous ambulatory peritoneal dialysis catheter insertion technique: a comparative study of percutaneous versus surgical insertion. Indian J Nephrol.

[10] Shah N, Goswell A, Cuesta C, Lemech L, Katz I (2023). Comparing surgeon- and nephrologist-inserted Tenckhoff catheters: experience from a metropolitan centre in Sydney. Intern Med J.

[11] Sampathkumar K, Mahaldar AR, Sooraj YS, Ramkrishnan M, Ajeshkumar Ajeshkumar, Ravichandran R (2008). Percutaneous CAPD catheter insertion by a nephrologist versus surgical placement: a comparative study. Indian J Nephrol.

[12] Esagian SM, Sideris GA, Bishawi M (2021). Surgical versus percutaneous catheter placement for peritoneal dialysis: an updated systematic review and meta-analysis. J Nephrol.

[13] Xie J, Kiryluk K, Ren H (2011). Coiled versus straight peritoneal dialysis catheters: a randomized controlled trial and meta-analysis. Am J Kidney Dis.

[14] Chow KM, Wong SS, Ng JK (2020). Straight versus coiled peritoneal dialysis catheters: a randomized controlled trial. Am J Kidney Dis.

[15] Singh V, Mishra SC, Singh P, Rout BB (2023). The influence of peritoneal dialysis catheter tip design on technique survival: a retrospective observational study. Indian J Nephrol.

